# Double-lumen catheter in the right jugular vein induces two sub-endothelial abscesses in an unusual place, the transition between the superior vena cava and the right atrium: a case report

**DOI:** 10.1186/1755-7682-7-37

**Published:** 2014-07-31

**Authors:** João Kennedy Teixeira Lima, Sylvia Rannyelle Teixeira Lima, Antonio Leonel de Lima Jr, Cícero Valdizébio Pereira Agra, Vitor Engrácia Valenti, Rayana Loch Gomes, Luciano Miller Rodrigues, João Antonio Correa, Rodrigo D Raimundo, Luiz Carlos de Abreu

**Affiliations:** 1 Unidade Acadêmica Ciências da Vida, Universidade Federal de Campina Grande, Cajazeiras, PB, Brasil; 2 Departamento de Cirurgia da Faculdade de Medicina do ABC, Santo André, SP, Brasil; 3 Laboratório de Delineamento de Estudos e Escrita Científica, Departamento de Saúde da Coletividade, Disciplina de Metodologia Científica, Faculdade de Medicina do ABC, Av. Príncipe de Gales, 821, 09060-650 Santo André, SP, Brazil; 4 Programa de Pós-Graduação em Fisioterapia, Faculdade de Ciências e Tecnologia, UNESP, Rua Roberto Simonsen, 305, 19060-900 Presidente Prudente, SP, Brazil

**Keywords:** Endocarditis, Central venous catheters, Renal dialysis, Hospitalisation

## Abstract

Endocarditis is a type of infection that is common in internal medicine wards and in haemodialysis clinics. The location that is most affected are the heart valves. Herein, we report a case of an uncommon abscess, a sub-endothelial abscess between the transition of the superior vena cava and the right atrium. There were several emboli to the lung and foot, and the agent was related to *Staphylococcus aureus* and a double-lumen catheter. Usually, this type of abscess is located in valves, either the tricuspid valve if related to catheters or injection drug use or the mitral valve if related to other causes. An exhaustive review was made, but we found no information about the location of this abscess and the rarity of the event motivating the report of infection.

## Introduction

If untreated, Infective Endocarditis (IE) is a fatal disease. IE is an endovascular, microbial infection of the intracardiac structures facing the blood, including infections of the large intrathoracic vessels and of intracardiac foreign bodies. The early characteristic lesion is variably sized, although destruction, ulceration or abscess formation may be seen earlier by echocardiography [1]. Endocarditis of the right-side of the heart is uncommon by virtue of the low haemodynamic pressure and lack of isolated or significant right-sided valvular deformities, or without endothelial injury. Central venous catheters (CVC) are a reliable option for clinical situations requiring immediate vascular access for haemodialysis (HD), as in the case of patients with uraemia [2]. Also, these intracardiac devices can injure the endothelium and progress with embolic events when they are infected.

## Case report

MECL was a 49 year-old, unemployed, married female who was born and raised in Aracati, Ceará, Brazil. She was Catholic and presented with fever and lack of strength for 1 month. She had been diabetic for 19 years and suffered hypertension for 10 years; the terminal Chronic Renal Failure that led to renal replacement therapy (RRT). All procedures were approved by the Ethical committee in Research of School of Medicine of ABC and are in compliance with the Helsinki Declaration.

The renal replacement therapy (RRT) started 5 months previously and evolved, in about one and a half months, to intermittent chills not related to HD. One month ago, the patient developed asthenia and prostration. She was initially treated with Kefazol and blood cultures were requested. Even with the use of medication, the patient remained febrile and presented with an erythematous lesion on her left foot. Cellulite was seen, which initiated the use of vancomycin and amikacin, and she was admitted for further evaluation due to the state of toxaemia.

During hospitalisation, bullous cellulitis began to develop in the affected foot. Insulin, in the form of enalapril, was then used. Three attempts were performed creating an arteriovenous fistula without any success. There are important background for diabetes and hypertension, but not for nephropathy.

The patient lost 11 pounds in two weeks, and was asthenic, with paresthesia in her lower limbs, and a dry cough for four days of hospitalisation, which progressed to the first episode of dyspnoea of her life. She also reported foamy urine, was febrile, pale, dehydrated, disoriented, had a normal heart rhythm with tricuspid systolic murmur, tachycardia, diminished breath sounds at the base without noise, had extensive erythematous lesions with signs of inflammation, precise limits, bulla measuring 3×2 cm on average in the dorsal region of the left foot, and the presence of multiple petechiae in the pretibial right limb.

**Figure 1:**
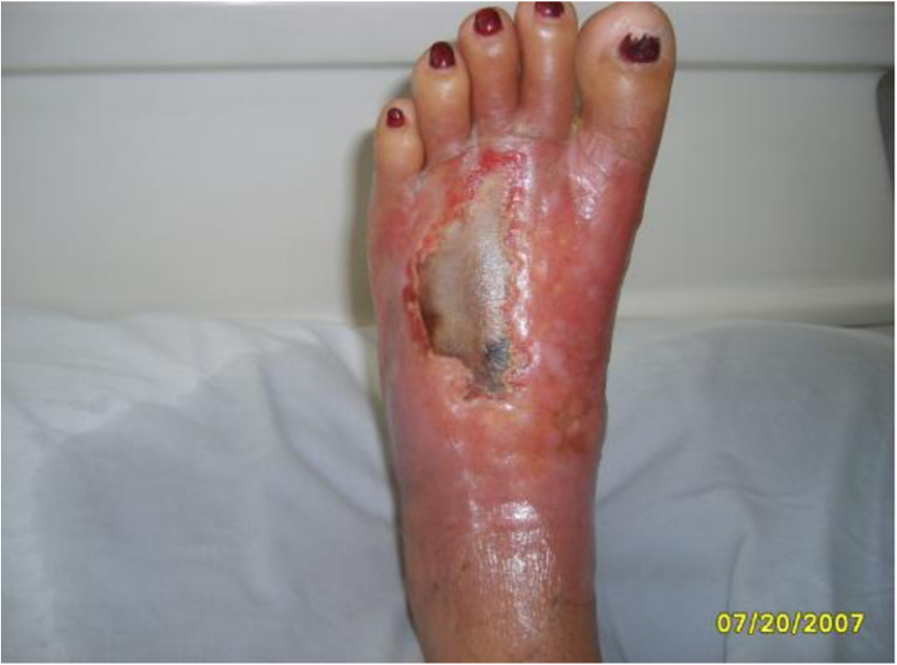
Hyperaemic and ulcerated lesion in the left lower limb.

**Figure 2:**
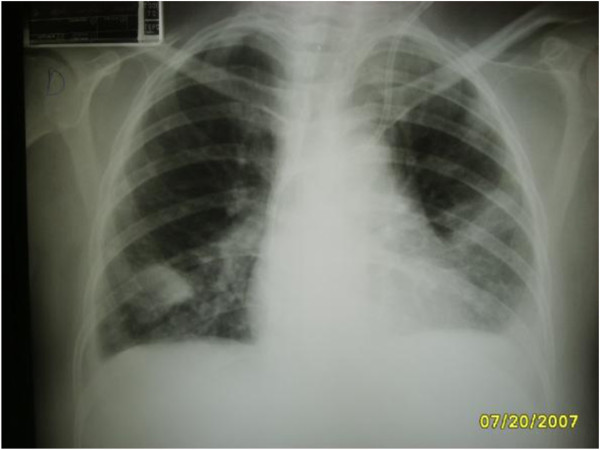
Image of chest radiography with areas of opacity and an image of double-lumen CVC for HD implanted in the left jugular vein.

**Figure 3:**
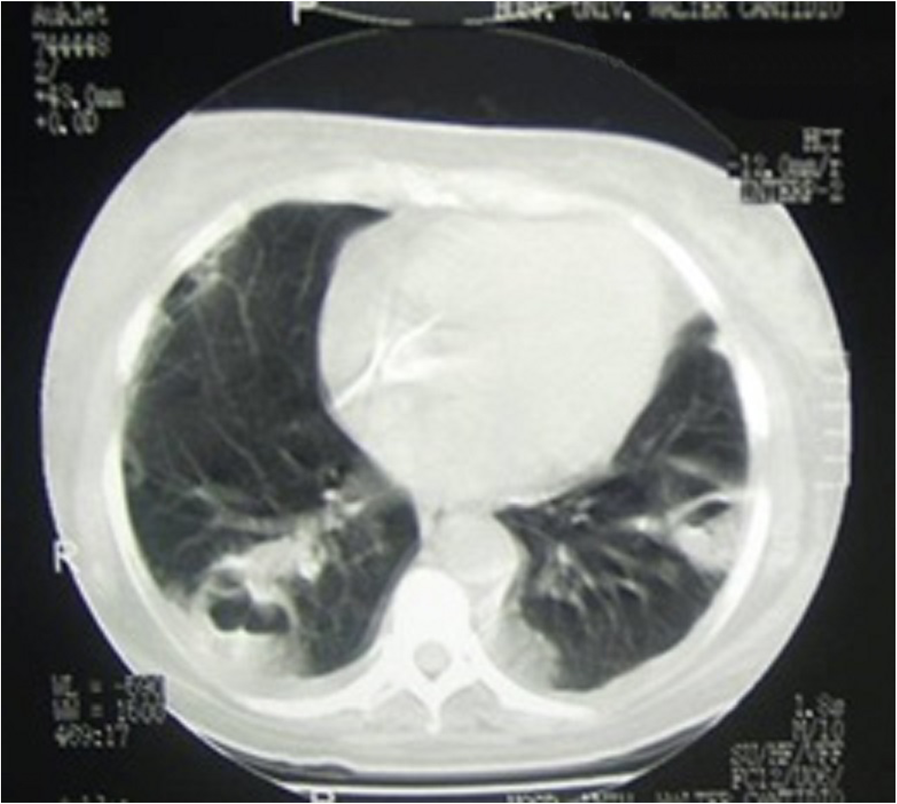
Computed tomography of the thorax with pulmonary lesions with air-fluid levels.

**Figure 4:**
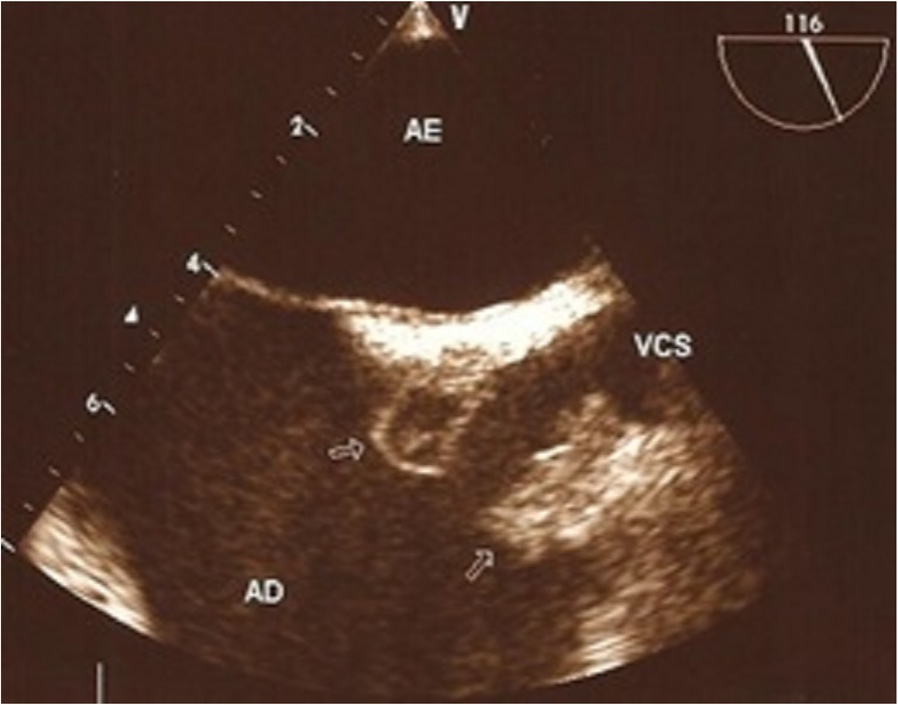
Echocardiography showing two images of sub-endothelial abscesses in the transition from the superior vena cava and right atrium.

Complementary tests identified an increased leukocyte count with a left shift, *Staphylococcus aureus* in the blood culture, and hypotransparency of the circular right and across the middle third of the left lung was seen by chest radiography. Transthoracic echocardiography (TTE) identified good ejection fraction, mild tricuspid insufficiency; concentric hypertrophy of the left ventricle, mitral flow compatible with left ventricle relaxation deficit; global and segmental contractility of preserved left ventricle; and mild pericardial effusion, absence of images compatible with vegetation. Due to the suspicion of endocarditis in right trans-oesophageal echocardiography (TEE) was requested, which surprisingly identified two areas of sub-endothelial abscesses in the transition between the superior vena cava and the right atrium. A lung tomography identified numerous lung abscesses. The patient died a few days after the diagnosis.Figure 1 presents hyperaemic and ulcerated lesion in the left lower limb, while Figure 2 indicates an image of chest radiography with areas of opacity and an image of double-lumen CVC for HD implanted in the left jugular vein.We observe in Figure 3 computed tomography of the thorax with pulmonary lesions with air-fluid levels and in Figure 4 we note the echocardiography showing two images of sub-endothelial abscesses in the transition from the superior vena cava and right atrium.

## Discussion

The highest rates of IE are observed among patients with prosthetic valves and catheters. Other risk factors include chronic rheumatic heart disease, age-related degenerative valvular lesions, HD, and coexisting conditions such as diabetes, human immunodeficiency virus infection, and intravenous drug use. The clustering of several of these predisposing factors with age probably explains the increased incidence of EI among persons aged 65 years or older [3-6].

The IE of the right side of the heart is uncommon by virtue of the low haemodynamic pressure and lack of isolated or significant right-sided valvular deformities [7].

IE, like most other syndromes of bacterial infection, has not escaped the impact of burgeoning antibiotic resistance among common pathogens [8-10].

The best form for diagnosis is the definition of Terms Used in the Modified Duke Criteria.

Definite IE:

Pathological criteria:

– Microorganisms demonstrated by culture or histological examination of a vegetation, a vegetation that has embolised, or an intracardiac abscess specimen; or

– Pathological lesions; vegetation or intracardiac abscess confirmed by histological examination showing active IE

Clinical criteria:

– 2 major criteria; or 1 major criterion and 3 minor criteria; or 5 minor criteria;

Possible IE:

– 1 major criterion and 1 minor criterion; or 3 minor criteria;

Rejected:

– Firm alternative diagnosis explaining evidence of IE; or Resolution of IE syndrome with antibiotic therapy for ≤4 days; or no pathological evidence of IE at surgery or autopsy, with antibiotic therapy for ≤4 days; or does not meet the criteria for possible IE outlined above.

The major criteria are:

– Blood culture positive for IE; typical microorganisms consistent with IE from 2 separate blood cultures: *Viridans streptococci*, *Streptococcus bovis*, HACEK group, *Staphylococcus aureus*; or

– Community-acquired enterococci in the absence of a primary focus; or

– Microorganisms consistent with IE from persistently positive blood cultures defined as follows: At least 2 positive cultures of blood samples drawn >12 h apart; or all 3 or a majority of ≥4 separate cultures of blood (with first and last sample drawn at least 1 h apart).

– Single positive blood culture for *Coxiella burnetii* or anti–phase 1 IgG antibody titre >1:800;

– Evidence of endocardial involvement: Echocardiogram positive for IE (TEE recommended for patients with prosthetic valves, rated at least “possible IE” by clinical criteria, or complicated IE [paravalvular abscess]; transthoracic echocardiography (TTE) as first test in other patients) defined as follows: oscillating intracardiac mass on valve or supporting structures, in the path of regurgitant jets, or on implanted material in the absence of an alternative anatomic explanation; or abscess; or new partial dehiscence of prosthetic valve; new valvular regurgitation (worsening or changing or pre-existing murmur not sufficient);

The minor criteria are:

– Predisposition, predisposing heart condition, or injecting drug user, fever, temperature >38°C; vascular phenomena, major arterial emboli, septic pulmonary infarcts, mycotic aneurysm, intracranial haemorrhage, conjunctival haemorrhages, and Janeway’s lesions;

– Immunologic phenomena: glomerulonephritis, Osler’s nodes, Roth’s spots, and rheumatoid factor;

– Microbiological evidence: positive blood culture but does not meet a major criterion, as noted above, or serological evidence of active infection with organisms consistent with IE [11-13]^.^.

Vascular access infections can lead to sepsis, IE, and metastatic infectious foci, and account for up to 10% of deaths in HD patients [14].

Systemic embolisation occurs in 22% to 50% of cases of IE. Emboli often involve major arterial beds, including the lungs, coronary arteries, spleen, bowel, and extremities. Moreover, the latter study reemphasised the increased risk of embolisation with increasing vegetation size during therapy, mitral valve involvement, and staphylococcal cause [15]. Of note, 2 independent studies have confirmed that the rate of embolic events dropped dramatically during or after the first 2–3 weeks of successful antibiotic therapy. In a study from 1991, the embolic rate dropped from 13 to <1.2 embolic events per 1000 patient-days during that time. In a more recent study, Vilacosta et al. confirmed the reduced frequency of embolisation after 2 weeks of therapy.

Moreover, the latter study reemphasised the increased risk of embolisation with increasing vegetation size during therapy, mitral valve involvement, and staphylococcal causes [9]. Infectious complications including catheter-related bloodstream infections (CRBSI) occurred at the frequency of 0.9-2.0 episodes/patient-year among the HD population, 10% of whom would be hospitalised with sepsis or metastatic infection (such as septic thrombosis, endocarditis and septic arthritis) [16]. Although CRBSI is very common, septic catheter-related pulmonary emboli are unusual, more so in the absence of endocarditis or infected right atrial thrombus.

*S. aureus* is the most common cause of disease in much of the developed world. This increase is primarily a consequence of healthcare contact (e.g., intravascular catheters, surgical wounds, indwelling prosthetic devices). In non-addicts, endocarditis arising from *S. aureus* primarily involves the left side of the heart and is associated with mortality rates ranging from 25% to 40%. *S. aureus* endocarditis in injecting drug users often involves the tricuspid valve. Cure rates for right-sided *S. aureus* IE in injecting drug users are high (>85%) and may be achieved with relatively short courses of treatment (<4 weeks).

The treatment can be realised with vancomycin for more than six weeks associated with gentamycin for two weeks [17]. The management of CVC-related bacteraemia requires prolonged antibiotic courses [14].

There are no reports of sub-endothelial abscesses between transition to the superior vena cava and right atrium, especially as it relates to HD catheters.

The aim is to remove CVC as quickly as possible, despite attempts to preserve vascular access, when it is related to HD. The CVC can be salvaged in two thirds of cases, although the bacteraemia may recur as long as 6 months later. However, the CVC should be removed in patients with symptoms of sepsis, and those with *S. aureus* bacteraemia [14].

During hospitalisation, there were several major and minor criteria for Duke to define the diagnosis of IE. Appropriate therapy was performed without success. The CVC was removed and initiated using Amikacin Vancomycin and corrected for renal function. The patient was very weak, there were several criteria of gravity, and the disease evolved, ultimately resulting in death in the Intensive Care Unit.

Mortality rates attributable to vascular access related to blood stream infections (VRBSI) among long-term HD patients vary from 12–25.9%. The type of vascular access determines the quality of life and the cost of dialysis treatment among end stage renal disease patients [18].

## Discussion

Thus, health care is conventionally regarded as the diagnosis, treatment, and prevention of disease, illness, injury, and other physical and mental impairments in humans. How we define the quality of public health at any given time must be compatible with future generations enjoying health in an equivalent way. Public health practitioners must also integrate sustain ability in the definition of public health [19]. Finally, Health is a Sustainable State, that is something constantly on the move and depending on constant attention, active maintenance and care.

## Consent

Written informed consent was obtained from the patient’s guardian/parent/next of kin for the publication of this report and any accompanying images.

## Competing interests

The authors declare that they have no competing interests.

## Authors’ contributions

JKTL, SRTL, ALL, CVPA, VEV, RLG, LMR, JAC, RDR and LCA. LCA, JKTL, RLG, JAC and VEV performed the statistical analysis. All authors revised the final version. All authors read and approved the final manuscript.
